# Efficient Facilitated Transport Polymer Membrane for CO_2_/CH_4_ Separation from Oilfield Associated Gas

**DOI:** 10.3390/membranes11020118

**Published:** 2021-02-07

**Authors:** Chunwei Zhang, Menglong Sheng, Yaoqiang Hu, Ye Yuan, Yulong Kang, Xiao Sun, Tao Wang, Qinghua Li, Xisen Zhao, Zhi Wang

**Affiliations:** 1 Research Institute of Yanchang Petroleum (Group) Co., Ltd., Yanchang Petroleum Science and Research Center, Xi’an 716000, China; zhangchunwei@sxycpc.com (C.Z.); yao.hu@stu.xjtu.edu.cn (Y.H.); kangyulong@sxycpc.com (Y.K.); sunxiao3882@sxycpc.com (X.S.); cheryl_vi@yeah.net (T.W.); 2 Chemical Engineering Research Center, School of Chemical Engineering and Technology, Tianjin University, Tianjin 300350, China; upcsml@163.com (M.S.); tjuyuanye@tju.edu.cn (Y.Y.); liqhua@tju.edu.cn (Q.L.)

**Keywords:** oilfield associated gas, CO_2_ capture, multilayer composite membrane, facilitated transport, CO_2_/CH_4_ separation

## Abstract

CO_2_ enhanced oil recovery (CO_2_-EOR) technology is a competitive strategy to improve oil field economic returns and reduce greenhouse gas emissions. However, the arbitrary emissions or combustion of the associated gas, which mainly consists of CO_2_ and CH_4_, will cause the aggravation of the greenhouse effect and a huge waste of resources. In this paper, the high-performance facilitated transport multilayer composite membrane for CO_2_/CH_4_ separation was prepared by individually adjusting the membrane structure of each layer. The effect of test conditions on the CO_2_/CH_4_ separation performance was systematically investigated. The membrane exhibits high CO_2_ permeance of 3.451 × 10^−7^ mol·m^−2^·s^−1^·Pa^−1^ and CO_2_/CH_4_ selectivity of 62 at 298 K and 0.15 MPa feed gas pressure. The cost analysis was investigated by simulating the two-stage system. When the recovery rate and purity of CH_4_ are 98%, the minimum specific cost of separating CO_2_/CH_4_ (45/55 vol%) can be reduced to 0.046 $·Nm^−3^ CH_4_. The excellent short-to-mid-term stability indicates the great potential of large industrial application in the CH_4_ recovery and CO_2_ reinjection from oilfield associated gas.

## 1. Introduction

The energy and environmental crisis are the key issues for sustainable development in the world today. With the continuous progress of the economy and society, energy demand is increasing year by year. Compared with traditional fossil energy, the large-scale promotion of renewable energy such as wind energy, solar energy, and biomass energy is still facing greater difficulties. The contradiction between energy supply and demand has become more severe. Therefore, the production capacity of fossil energy needs to be expanded urgently [1].

Abundant low-permeability oil and gas resources are buried in petroliferous basins dominated by continental deposits [2]. According to incomplete statistics, Yanchang Oilfield is the second-largest low-permeability oilfield in China [3], with proven reserves of 1.12 × 10^8^ t and an oil-bearing area of 215.5 km^2^ [4]. However, most oil fields in this area are currently in the late stage of water-enhanced oil recovery. The high-water content and low pressure of the oil reservoirs lead to reduced oil production and poor production efficiency.

In recent years, CO_2_ enhanced oil recovery (CO_2_-EOR) technology has faced rapid development and has been widely used in many oil and gas fields in the world [5,6,7,8]. According to statistics, compared with other types of oil displacement technology, CO_2_-EOR could increase oil and gas recovery by 8–25% [9]. CO_2_ miscible flooding is a very effective way to improve oil recovery, which is achieved by injecting supercritical or liquid CO_2_ into the reservoir to reach the minimum miscibility pressure (MMP) and blended with crude oil [10]. Crude oil miscible with CO_2_ is greatly swelled, reducing its viscosity and interfacial tension, thereby improving the fluidity of crude oil [11]. In addition, the production pressure in the mining well can be also increased [12]. Therefore, the oil and gas recovery rate has been greatly improved. Besides, CO_2_-EOR technology is also an important way to realize carbon capture and storage (CCS) [13]. CO_2_-EOR technology can store CO_2_ gas geologically in oil and gas reservoirs for a long time, thus it can simultaneously realize the social benefits of CO_2_ storage and the economic benefits of CO_2_ flooding [5,14,15].

However, when CO_2_ is used to enhance oil recovery, a large amount of oilfield associated gas containing CO_2_ and light hydrocarbons (mainly CH_4_) will also be discharged [16]. The flow rate and CO_2_ content of the oilfield associated gas fluctuate greatly with time, which cannot be reinjected, stored, and utilized directly [17]. In the initial stage of CO_2_-EOR, the oilfield associated gas features a low flow rate, low concentration of CO_2_ (~5 vol%), and high concentration of CH_4_ (~75 vol%) [18]. While in the advanced stage of CO_2_-EOR, the CO_2_ content gradually increases, and finally stabilizes at 60~80 % [19]. Due to the high CO_2_ concentration, the oilfield associated gas cannot be directly burned. Direct emptying will cause a large amount of greenhouse gas emissions and a waste of resources, while reinjecting associated gas directly to enhance oil recovery does not meet the basic requirement that the minimum miscibility pressure must be lower than the reservoir pressure. In addition, the oilfield associated gas also exhibits obvious features of low pressure and considerable flux fluctuations. Thus, the traditional separation methods always require a lot of energy to increase the associated gas pressure, which is less economical. Therefore, it is necessary to develop a CO_2_ separation technology with low operating cost, high separation flexibility, and suitable for separating low-pressure mixed gas to realize the on-site separation of oilfield associated gas.

In this paper, a high-performance composite membrane for CO_2_ separation of oilfield associated gas was prepared by optimizing membrane structure layer by layer. The influence of feed gas humidity, temperature, composition, and pressure on membrane separation performance were further investigated to explore the optimal operating conditions of composite membranes. In addition, a secondary membrane separation process utilizing this membrane was simulated to obtain the lowest operating cost and ideal operating conditions.

## 2. Materials and Methods

### 2.1. Materials

Polydimethylsiloxane (PDMS) prepolymer was supplied from Shin-Etsu Chemical Co., Ltd. (Tokyo, Japan). Ethyl orthosilicate (TEOS, 99 %), dibutyltin dilaurate (DBD, 99 %), sodium polyacrylate (PAAS, Mw = 15 kDa), Poly (vinyl alcohol) 1799 (PVA 1799) and sodium dodecyl sulfate (SDS, AR) were purchased from Aladdin Reagent Co., Ltd. (Shanghai, China). N-heptane (98.5 %) was bought from Tianjin Bohua Chemical Reagents Co., Ltd. (Tianjin, China). Polyvinylamine (PVAm) was synthesized through radical polymerization according to our previous work [20]. Deionized water with a conductivity of less than 5 μS·cm^−1^ was prepared by reverse osmosis system. The polysulfone (PSf) ultrafiltration membrane with a molecular weight cut-off at 45 kDa was obtained from Jozzon Membrane Technology Co., Ltd. (Dongying, Shandong, China).

### 2.2. Methods

#### 2.2.1. Preparation of Multilayer Composite Membranes

According to the previous work [21], the cross-linked PDMS was prepared by the cross-linking reaction of hydroxyl-terminated PDMS with TEOS under the catalysis of DBD at 308 K and saturated humidity. The gutter layer was prepared by the wet-dry combined coating method. That is, the cross-linked PDMS was coated with the wet coating thickness of 300 μm on the PSf ultrafiltration membrane that possesses no obvious water on the surface but abundant water in the internal pore channels, so that the PDMS solution could not infiltrate into the pore of PSf ultrafiltration membrane, avoiding the excessive drop in gas permeance.

The main component of the solution utilized for coating the selective layer is PVAm, while the remaining ingredients include 20 mol% PVA, 1 mol% PAAS, and 10 mol% SDS, which was shortened to PPPS in this paper. The additives in the PPPS solution were mainly used to enhance the CO_2_ separation ability and interlayer compatibility [22]. The PPPS/PDMS/PSf composite membranes were manufactured by coating PPPS solution directly on the dried PDMS gutter layer with a wet coating thickness of 150 μm. The wet membranes were placed and dried in a constant environment with the temperature and humidity of 303 K and 40 %RH respectively for subsequent tests.

#### 2.2.2. Characterization

**Figure 1:**
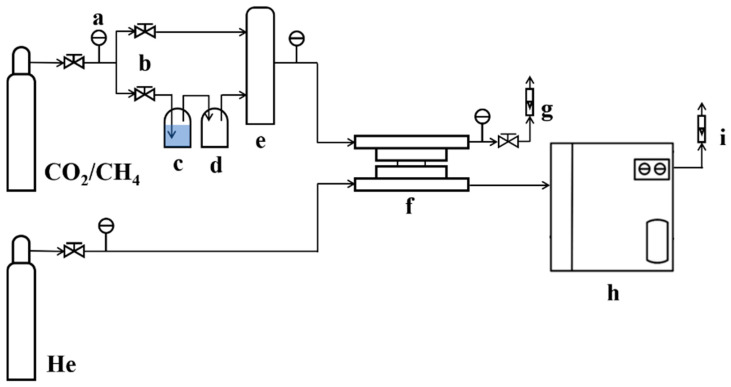
Schematic diagram of the laboratory-made gas permeance analysis platform. The letters in Figure 1 represent: (**a**) high-precision pressure gauge; (**b**) high-precision needle valve; (**c**) humidification tank; (**d**) dehumidification tank; (**e**) buffer tank; (**f**) membrane cell; (**g**) mass flowmeter; (**h**) gas chromatography; (**i**) soap film gas meter.

The cross-section topographies of the PDMS/PSf and PPPS/PDMS/PSf composite membranes were screened by scanning electron microscopy (SEM, Nova NanoSEM 430, FEI, Hillsboro, OR, USA). The functional groups of the membranes were evaluated by Fourier Transform Infrared (FTIR) spectroscopy (FTS-6000, Bio-Rad, Hercules, CA, USA). The CO_2_/CH_4_ mixed gas separation performance was estimated by the laboratory-made gas permeance analysis platform. As shown in Figure 1, the prepared CO_2_/CH_4_ mixed gases with different CO_2_ concentrations served as the feed gas flow into membrane cell at a set pressure. The CO_2_-rich penetrate gas driven by He was analyzed in the gas chromatograph (7890B, Agilent, Palo Alto, CA, USA) with a certain flow rate. The ratio of humidified gas (saturated) and dry gas in the feed gas can be controlled by adjusting the precision needle valve, thereby controlling the relative humidity of the feed gas. All error bars represented the standard errors of the performance of three membranes prepared under the same conditions.

#### 2.2.3. Performance Assessment

The CO_2_/CH_4_ separation performance of the composite membranes was evaluated by two main parameters, permeance, and selectivity. Permeance indicates how fast the gas passes through the membrane, which can be calculated by the following formula:

(1) $$R_{i}=\frac{Q_{i}}{\Delta p_{i}A}$$

where, *Q*_i_ is defined as the flow rate of gas composition (CO_2_ or CH_4_) penetrating through the membrane at the steady-state, the unit of which used in this paper is mol·s^−1^·Pa^−1^; ∆*p*_i_ stands for the partial pressure difference of the gas composition between the retentate and the permeate side of the membrane; *A* is the effective separation area of the membrane. Under ideal circumstances, the CO_2_/CH_4_ selectivity of the membrane is approximately equal to:

(2) $$\alpha =\frac{R_{i}}{R_{j}}$$

## 3. Results

### 3.1. Preparation of High-Performance CO_2_ Separation Composite Membrane

#### 3.1.1. PDMS Gutter Layer

PDMS is the most widely used gutter layer material, the thickness of which is directly related to the gas permeability of the composite membrane [23,24]. The concentration of PDMS solution determines the thickness of the gutter layer. Therefore, in this section, the adjustment of the gutter layer thickness was achieved by fixing the wet coating thickness (300 μm) and changing the concentration of the cross-linked PDMS solution. To select the optimal PDMS concentration, the CO_2_/CH_4_ mixed gas (45/55 vol%) separation performance was examined at 298 K, saturated humidity and 0.15 MPa feed gas pressure after coating 0.15 wt% PPPS solution with a wet coating thickness of 150 μm on the prepared PDMS/PSf composite membranes.

**Figure 2:**
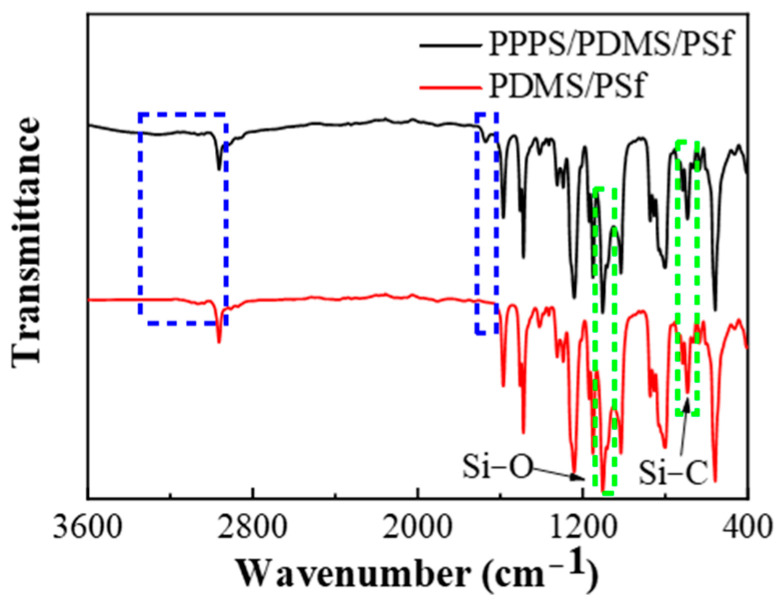
The infrared spectrums of Polydimethylsiloxane (PDMS)/ polysulfone (PSf) and PPPS/PDMS/PSf composite membranes.

The infrared spectrum of PDMS/PSf and PPPS/PDMS/PSf composite membranes were implemented to characterize the functional groups of both membranes. As presented in Figure 2, the absorption peaks at 690 cm^−1^ and 1100 cm^−1^ are the characteristic bands of Si-C and Si-O respectively, indicating the successful coating of PDMS on PSf substrate. However, due to the thin PPPS separation layer, the infrared spectrum of the PPPS/PDMS/PSf composite membrane contains all the absorption bands of PDMS. The absorption bands around 3300 cm^−1^–3000 cm^−1^ and 1670 cm^−1^ confirm the presence of –OH and –NH_2_, which proves the PPPS/PDMS/PSf composite membranes were successfully prepared.

**Figure 3:**
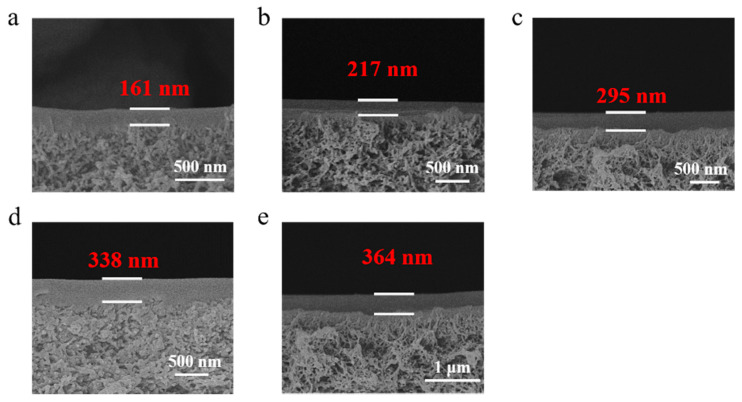
The cross-section topographies of the PPPS/PDMS/PSf composite membranes with different PDMS concentrations. The letters a~e respectively represent the concentration of PDMS used in membranes: 0.5 wt% (**a**), 1.0 wt% (**b**), 1.5 wt% (**c**), 2.0 wt% (**d**), and 2.5 wt% (**e**). The wet coating thickness is 300 μm.

Figure 3 shows the cross-section topographies of the PPPS/PDMS/PSf composite membranes prepared based on the gutter layers with the PDMS concentration of 0.5–2.5 wt%. The continuous interfaces are clearly presented between the gutter layers and PSf substrate, indicating that the wet-dry combined coating method can effectively inhibit the pore penetration of PDMS in the polysulfone. The thickness of the composite membranes prepared by different PDMS concentrations were investigated further. As shown in Figure 3, with the increase of PDMS concentration, the thickness of PPPS/PDMS/PSf composite membranes increased from 161 nm to 364 nm.

**Figure 4:**
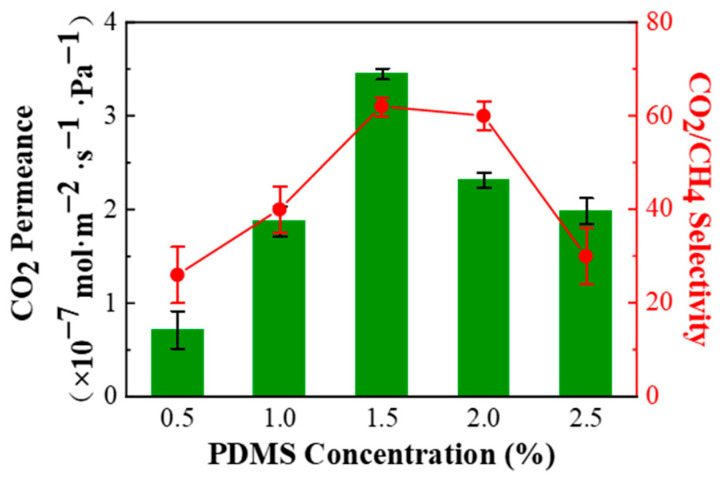
Effect of different PDMS concentrations on the CO_2_/CH_4_ (45/55 vol%) mixed gas separation performance of PPPS/PDMS/PSf composite membrane. Test conditions: 298 K, saturated humidity, and 0.15 MPa.

The CO_2_/CH_4_ (45/55 vol%) mixed gas separation performance of PPPS/PDMS/PSf composite membranes prepared from different PDMS concentrations is shown in Figure 4. The separation performance of the composite membranes increases first and then decreases with the increasing PDMS concentration. When the PDMS concentration reaches 1.5 wt%, the composite membrane possesses the best performance. The reasons are as follows. When the PDMS concentration is too low (≤1.0 wt%), the PDMS cannot completely cover the surface of the PSf substrate, which causes the PPPS solution to partially penetrating into the pores of the PSf substrate. According to the resistance model of the composite membrane, the total resistance of the composite membrane increases linearly with the increasing thickness of the polymer penetration area [25]. The incomplete membrane structure results in the fluctuating CO_2_ permeance and low CO_2_/CH_4_ selectivity. However, when the PDMS concentration increases to more than 2 wt%, the increasing thickness of the gutter layer increases the gas resistance, which reduces the gas permeance. Besides, the strong hydrophobicity of the prepared PDMS/PSf membranes affects the malleability of the PPPS solution, leading to the unevenness of the separation layer, and eventually increases the CH_4_ permeance. Thus, the CO_2_/CH_4_ selectivity was reduced.

#### 3.1.2. PPPS Separation Layer

With hydrophilic PVAm as the main ingredient, the PPPS solution achieves excellent compatibility between the hydrophilic separation layer and the hydrophobic PDMS surface by the synergistic effect of the surfactant and thickener [22]. Based on the optimal PDMS concentration determined in Section 3.1.1, the effect of the PPPS concentration on the CO_2_/CH_4_ separation performance of the PPPS/PDMS/PSf composite membrane was further explored fixing the wet coating thickness (150 μm) and changing the concentration of the PPPS solution.

**Figure 5:**
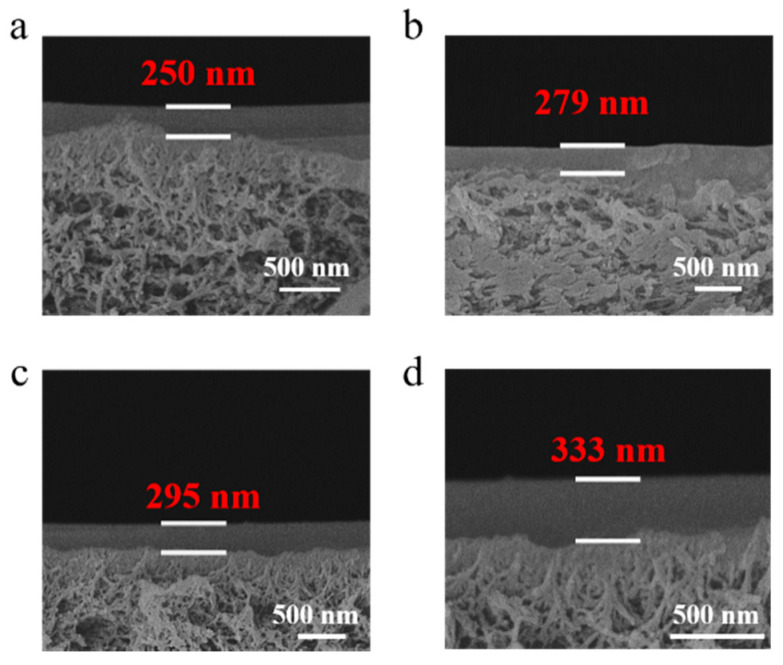
The cross-section topographies of the PPPS/PDMS/PSf composite membranes with different PPPS concentrations. The letters a~d respectively represent the concentration of PPPS used in membranes: 0.05 wt% (**a**), 0.10 wt% (**b**), 0.15 wt% (**c**), and 0.20 wt% (**d**). The wet coating thickness is 150 μm.

The cross-section topographies of the PPPS/PDMS/PSf composite membranes with the PPPS concentration between 0.05 wt% and 0.20 wt% were investigated by SEM. As shown in Figure 5, the thickness of the PPPS/PDMS/PSf composite membranes increases from 250 nm to 333 nm with the increasing PPPS concentration, which indicates that the separation layer is complete and thin.

**Figure 6:**
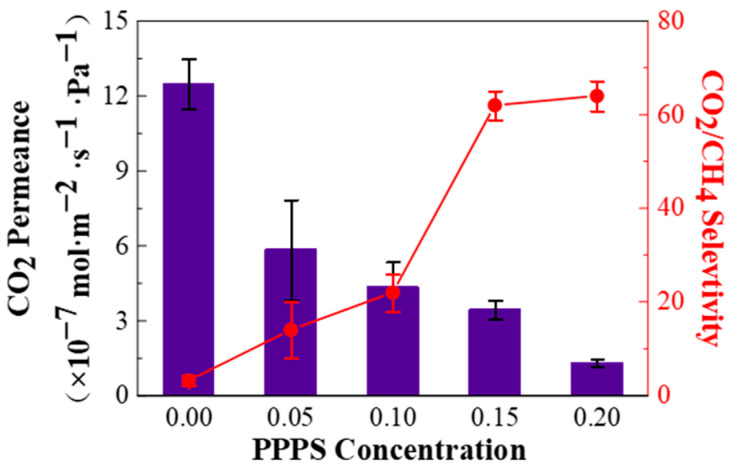
Effect of different PPPS concentrations on the CO_2_/CH_4_ (45/55 vol%) mixed gas separation performance of PPPS/PDMS/PSf composite membrane. Test conditions: 298 K, saturated humidity, and 0.15 MPa.

Figure 6 depicts the CO_2_/CH_4_ (45/55 vol%) mixed gas separation performance of PPPS/PDMS/PSf composite membranes prepared from different PPPS concentrations. As the PPPS concentration increases, the CO_2_ permeance gradually decreases, while the CO_2_/CH_4_ selectivity gradually increases. When the PPPS concentration is at 0.15 wt%, the PPPS/PDMS/PSf membrane exhibits the best separation performance that the CO_2_ permeance of 3.451 × 10^−7^ mol·m^−2^·s^−1^·Pa^−1^ and CO_2_/CH_4_ selectivity of 62. This is mainly due to the following reasons. When the PPPS concentration is less than 0.10 wt%), due to the low viscosity, the PPPS solution can hardly form an intact separation layer, resulting in the formation of defects without gas selectivity. While the increasing PPPS solution (>0.15 wt%) thickens the separation layer, which increases the gas resistance through the membrane.

### 3.2. Effect of Feed Gas Conditions on Separation Performance

#### 3.2.1. Relative Humidity of the Feed Gas

The effect of the relative humidity of the feed gas on CO_2_/CH_4_ (45/55 vol%) separation performance of the PPPS/PDMS/PSf composite membrane was explored under 298 K and 0.15 MPa feed pressure. The relative humidity of the feed gas was adjusted by controlling the opening of the two high-precision needle valves.

**Figure 7:**
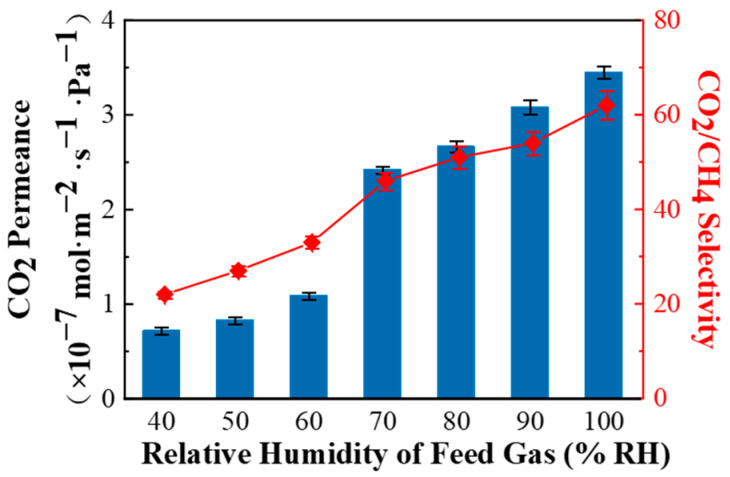
Effect of relative humidity of feed gas on the CO_2_/CH_4_ (45/55 vol%) mixed gas separation performance of PPPS/PDMS/PSf composite membrane. Test conditions: 298 K and 0.15 MPa.

As shown in Figure 7, with the increasing relative humidity of the feed gas, the CO_2_ permeance and CO_2_/CH_4_ selectivity of the PPPS/PDMS/PSf composite membrane also increase. This is due to the following two aspects. Firstly, PVAm with abundant primary amine groups, which is the dominance in the separation layer, can react specifically and reversibly with CO_2_ and promote the transfer of CO_2_ across the membrane. The mechanism of primary amine groups promoting CO_2_ transfer can be expressed as the following formula [26]:

(3) $${2\mathrm{RNH}}_{2}{+\mathrm{CO}}_{2}\overset{}{↔}{\mathrm{RNHCOO}}^{-}{+\mathrm{RNH}}_{3}{}^{+}$$

(4) $${\mathrm{RNH}}_{2}{+H}_{2}{O+\mathrm{CO}}_{2}\overset{}{↔}{\mathrm{HCO}}_{3}{}^{-}{+\mathrm{RNH}}_{3}{}^{+}$$

Due to a large number of active hydrogens, the primary amine groups can slowly react with CO_2_ without water. However, water can not only be used as reactants to participate in the CO_2_ facilitated transport, but also promote the ionization of primary amine groups and CO_2_, thereby accelerate the reversible reaction. Therefore, with the increasing relative humidity of feed gas, the positive effect of water on the reversible reaction improves, leading to an increase in CO_2_ permeance.

Secondly, water also promotes the CO_2_ adsorption on the membrane surface, while the adsorption capacity of CH_4_ on the membrane surface is reduced due to the competitive adsorption. Since CH_4_ penetrates through the solution-diffusion mechanism in the composite membrane, the permeation rate of CH4 will also decrease with the increase of relative humidity. Thirdly, as the relative humidity increases, PPPS with strong hydrophilic groups (amine and hydroxyl groups) adsorb and solve a lot of water, resulting in swelling and increase of the free volume of the composite membrane, and thus accelerate the permeance of CO_2_ and CH_4_ in the PPPS/PDMS/PSf composite membrane. Under the combined effect of the above, with the increasing relative humidity of the feed gas, CO_2_ permeance greatly increases while CH_4_ permeance slowly increases, which ultimately leads to an increase in the CO_2_/CH_4_ selectivity. Therefore, the PPPS/PDMS/PSf composite membranes are more suitable for use under high humidity conditions.

#### 3.2.2. Temperature of the Feed Gas

**Figure 8:**
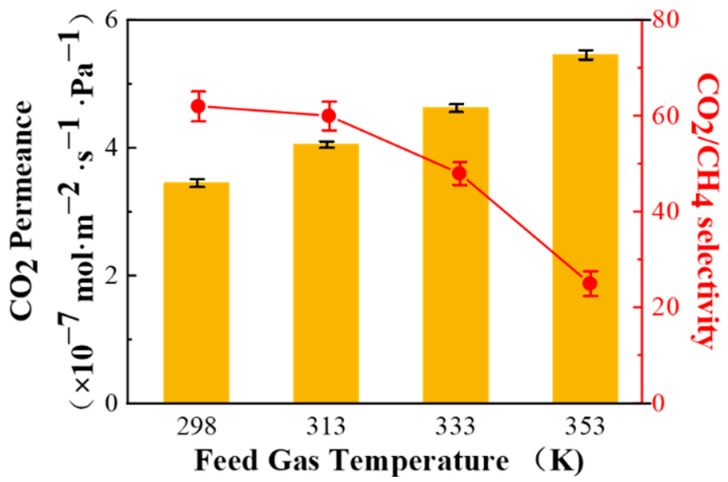
Effect of the feed gas temperature on the CO_2_/CH_4_ (45/55 vol%) mixed gas separation performance of PPPS/PDMS/PSf composite membrane. Test conditions: saturated humidity and 0.15 MPa.

The effect of the feed gas temperature on CO_2_/CH_4_ (45/55 vol%) separation performance of the PPPS/PDMS/PSf composite membrane was explored under saturated humidity and 0.15 MPa feed pressure. As shown in Figure 8, with the increasing feed gas temperature, the CO_2_ permeance of PPPS/PDMS/mPSf composite membrane gradually increases, and the CO_2_/CH_4_ selectivity reduces rapidly, which can be attributed to the following reasons.

Firstly, the saturated vapor pressure increased with the feed gas temperature, which causes the increase of moisture content in the feed gas. As mentioned in Section 3.2.1, the increase of the water content in the feed gas strengthens the swelling degree and free volume of the composite membrane, resulting in an increase in the gas permeance. In addition, the increase of water content in the composite membrane also enhances the CO_2_ facilitated transport. Secondly, as the feed gas temperature increases, due to the exothermically reversible reaction between CO_2_ and primary amine groups, the reaction rate increases but the forward reaction equilibrium constant decreases, which is not conducive to the CO_2_ facilitated transport. Thirdly, the solution and diffusion process of CH_4_ adheres to Arrhenius’ law [27,28], thus the CH_4_ permeance increases significantly with the increasing feed gas temperature.

In summary, with the increscent feed gas temperature, the CO_2_ and CH_4_ permeance in the PPPS/PDMS/mPSf composite membrane are both confronted with an upward trend. However, due to the multiple factors analyzed above, the CO_2_ permeance could increases more slowly, which ultimately leads to a decline in the CO_2_/CH_4_ selectivity. Thus, the ideal operating temperature of the PPPS/PDMS/PSf composite membrane is at 298 K.

#### 3.2.3. CO_2_ Concentration of the Feed Gas

**Figure 9:**
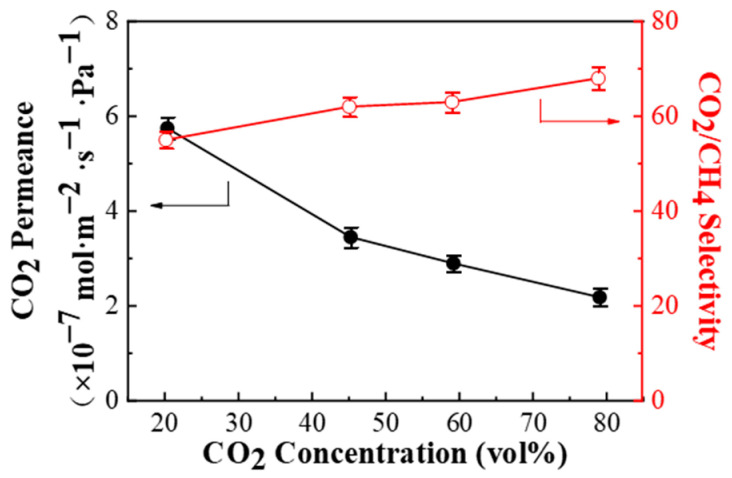
Effect of the feed gas CO_2_ concentration on the CO_2_/CH_4_ mixed gas separation performance of PPPS/PDMS/PSf composite membrane. Test conditions: 298 K, saturated humidity, and 0.15 MPa.

As the CO_2_ concentration of the oilfield associated gas changes greatly during the entire life of the oilfield, thus, it is especially significant to make a comprehensive study of the effect of the feed gas CO_2_ concentration on the CO_2_/CH_4_ separation performance of the PPPS/PDMS/PSf composite membranes. As presented in Figure 9, with the increasing CO_2_ concentration, the CO_2_ permeance keeps dropping, while the CO_2_/CH_4_ selectivity constantly grows. The main reasons are as follows.

According to the formula obtained by Zhang et al. [29], the CO_2_ permeance of the facilitated transport membranes can be defined by Formula (5).

(5) $$R_{A}=\frac{D_{A}}{lH_{A}}+\frac{D_{\mathrm{AX}}KC_{T}}{l\left(H_{A}+Kp_{A,0}\right)}$$

where *R*_A_ is the CO_2_ permeance, *p*_A,0_ is the CO_2_ partial pressure of the feed gas, *D*_A_ and *D*_AX_ are the diffusion coefficients of CO_2_ and CO_2_-carrier complexes respectively, *l* is the thickness of the membrane, *H*_A_ is the Henry coefficient of CO_2_, *K* is the equilibrium constant of the reversible reaction between CO_2_ and the carrier, *C*_T_ is the total concentration of the carrier. Therefore, the growing CO_2_ partial pressure in the feed gas leading to the contribution of facilitated transport to the total CO_2_ permeance decline. As for CH_4_, on the one hand, the solubility coefficient and diffusion coefficient of CH_4_ in the membrane decrease with the falling CH_4_ partial pressure [30]. On the other hand, due to the competitive adsorption, the amount of CH_4_ adsorbed on the membrane surface tends to downward with the increase of the CO_2_ partial pressure. To sum up, as the CO_2_ partial pressure in the feed gas increases, the CO_2_ permeance of the PPPS/PDMS/PSf facilitated transport membrane keeps decreasing, while the CH_4_ permeance drops faster, leading to the CO_2_/CH_4_ selectivity showing an upward trend.

#### 3.2.4. Pressure of the Feed Gas

**Figure 10:**
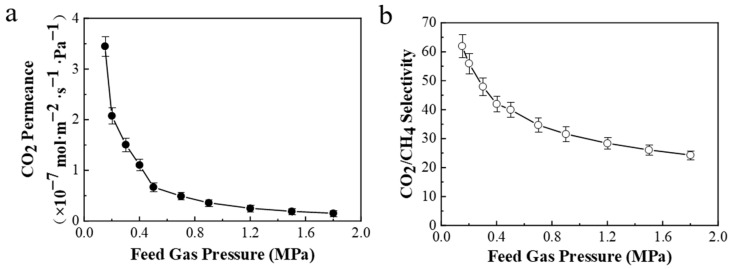
Effect of the feed gas pressure on the CO_2_/CH_4_ mixed gas separation performance of PPPS/PDMS/PSf composite membrane. (**a**), the pressure-dependent CO_2_ permeance of the PPPS/PDMS/PSf membrane. (**b**), the correspond-ing CO_2_/CH_4_ selectivity of the PPPS/PDMS/PSf membrane varying with feed pressure. Test conditions: 298 K and saturated humidity.

To further examine the effect of feed gas pressure on CO_2_/CH_4_ (45/55 vol%) separation performance, the PPPS/PDMS/PSf composite membrane was tested at a feed pressure of 0.15~1.8 MPa. As shown in Figure 10, the CO_2_ permeance and CO_2_/CH_4_ selectivity decrease rapidly with increasing feed gas pressure, which is mainly due to the following reasons. First of all, PVAm is a typical material mainly utilized in facilitated transport membrane, thus the PPPS/PDMS/PSf composite membrane displays the representative characteristics of facilitated transport, that is, CO_2_ permeance decreases with increasing CO_2_ partial pressure [29,31]. In addition, CH_4_ penetrates through the composite membrane by solution-diffusion mechanism, causing the CH_4_ permeance to be almost unchanged. Therefore, the CO_2_/CH_4_ selectivity decreases as the feed gas pressure increases.

### 3.3. Separation Process Simulation under Actual Working Conditions

#### 3.3.1. System and Simulation Condition Setting

To investigate the industrial potential of the PPPS/PDMS/PSf composite membranes, a two-stage membrane process was designed for the CO_2_ separation and CH_4_ recovery from the oilfield associated gas, which is the most economical separation process in the case of high product purity and recovery [32,33,34]. In this simulation, the CH_4_ purity (98 vol%) and CH_4_ recovery rate (98%) were chosen as the separation requirements [35,36]. The feed pressure of the two-stage membrane process was optimized when the gas composition of raw gas was 45 vol% CO_2_ and 55 vol% CH_4_.

**Figure 11:**
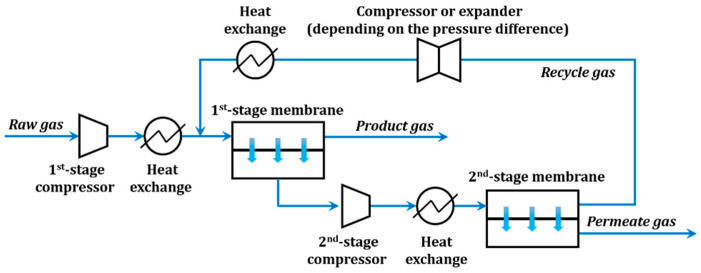
Schematic diagram of a two-stage membrane process.

The two-stage membrane separation process is shown in Figure 11. The flow rate of raw gas is set as 1000 Nm^3^·h^−1^, and the pressure is 0.15 MPa (absolute pressure, the same below). After being compressed by the compressor, the raw gas encountered with the recycle gas was separated by the 1st-stage membrane. The retentate gas (CH_4_ concentration at 98 vol%) from the 1st-stage was stored for further purification, while the permeate gas was recompressed and divided by the 2nd-stage membrane into the recycle gas and the penetrate gas rich in CO_2_. The feed pressures of the 1st- and 2nd-stages are in the range of 0.3~1.5 MPa, and the pressures on the permeate side are set as 0.1 MPa.

The gas separation process was described by the cross-flow model [37,38] and computed by MATLAB software, according to previous work [39,40]. Detailed information on the mathematical model is provided in S2 (Supplementary Information).

#### 3.3.2. Simulation of the Total Membrane Area and Specific Electricity Consumption

**Figure 12:**
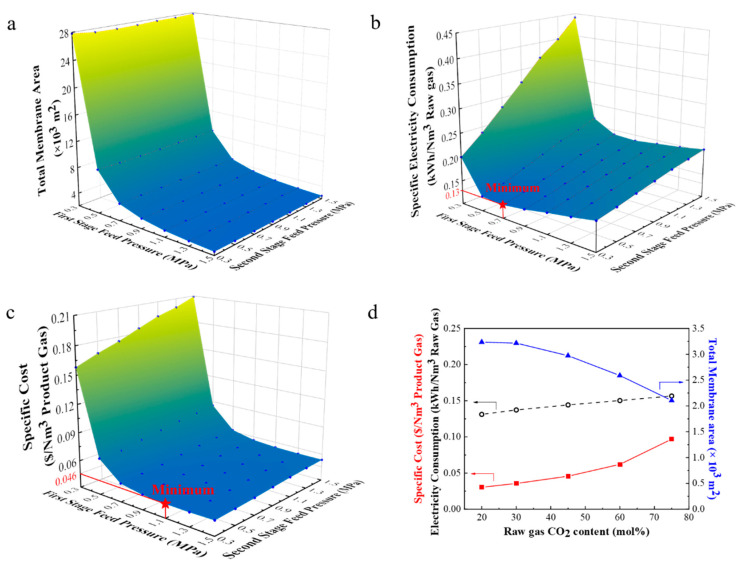
Simulation results of a two-stage membrane separation process using PPPS/PDMS/PSf composite membranes. Impact of the 1st- and 2nd-stage feed pressures on the total membrane area (**a**), the specific electricity consumption (**b**), and the specific cost (**c**). (**d**) Impact of CO_2_ concentration on the specific cost, the specific electricity consumption, and the total membrane area, when the 1st- and 2nd-stage feed pressures are 1.1 and 0.3 MPa respectively.

The total membrane area required to complete the set conditions was simulated by adjusting the feed pressure of the 1st-stage membrane and the 2nd-stage membrane separately. As shown in Figure 12a, with the increase of the 1st-stage feed pressure, the total membrane area decreases significantly, while the 2nd-stage feeding pressure has little influence on the total membrane area. The reason is that the gas processing capacity of the 1st-stage membrane process is much higher than that of the 2nd-stage. With the increasing feed gas in the 1st-stage, the gas flux per membrane area also increases significantly, thus the membrane area decreases dramatically.

However, although the increase in the feed pressure can reduce the membrane area, the energy consumption to compress the gas is relatively increased. As shown in Figure 12b, when the 1st-stage feed pressure is fixed, with the increasing 2nd-stage feed pressure, the specific electricity consumption of the compressor increases. When fixing the 2nd-stage feed pressure, the increasing 1st-stage feed pressure leads to the specific electricity consumption first decreasing and then increasing. Therefore, when the 1st- and 2nd-stage feed pressures are 0.7 MPa and 0.3 MPa respectively, the minimum specific electricity consumption can be reduced to 0.13 kWh/Nm^3^ raw gas.

#### 3.3.3. Simulation of the Effect of CO_2_ Concentration on Specific Cost

The specific cost of the two-stage process using PPPS/PDMS/PSf composite membranes was calculated mainly referring to the method mentioned in the previous papers [39,41]. The system operation time was set as 8000 h per year. The membrane module cost was considered as 50 $·m^−2^ [36], the referenced fixed investment cost was selected as 394,000$ per 2000 m^2^ membranes [42] and the electricity cost was consulted as 0.1 $·kWh^−1^ [32,43]. In brief, the total annual cost is the sum of the investment cost, the cost for annual operating and maintenance, and energy cost. Detailed information on the economic evaluation is provided in S3 (Supplementary Information).

As presented in Figure 12c, when the raw gas was fixed as 45 vol% CO_2_ and 55 vol% CH_4_, the minimum specific cost can be reduced to 0.046 $·Nm^−3^ CH_4_ with the 1st- and 2nd-stage pressures of 1.1 MPa and 0.3 MPa, respectively. Compared with the 2nd-stage feed pressure, the 1st-stage feed pressure exerts a greater influence on the specific cost, for the 1st-stage feed pressure affects the total membrane area more obviously.

However, CO_2_ concentration varies greatly in the life of oilfield associated gas. Based on the above pressures, the specific cost was further simulated by changing the CO_2_ concentration. As shown in Figure 12d, with the increase of CO_2_ concentration in the raw gas, the required membrane area gradually decreases. Simultaneously, the product flow rate (98 vol% CH_4_) declines, but the flow rate of the gas that needs to be compressed increasingly augment. Thus, when CO_2_ concentration is at 20 vol%, the specific cost is 0.030 $·Nm^−3^ CH_4_, while when CO_2_ concentration reaches up to 75 vol%, the specific cost gradually rises to 0.097 $·Nm^−3^ CH_4_.

### 3.4. Short-to-Mid-Term Stability of PPPS/PDMS/PSf Composite Membrane

**Figure 13:**
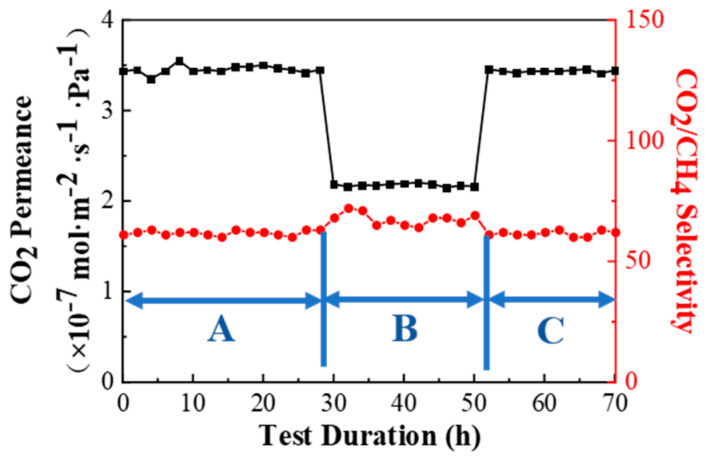
Short-to-mid-term stability of PPPS/PDMS/PSf composite membrane. Test conditions: 298 K, saturated humidity, and 0.15 MPa.

The PPPS/PDMS/PSf composite was continuously tested at 298 K, saturated humidity, and 0.15 MPa of the feed gas pressure for 70 h to verify the short-to-mid-term CO_2_/CH_4_ separation performance. As shown in Figure 13, the short-to-mid-term stability test was divided into three stages. The feed gas in the first 30 h consisted of 45 vol% CO_2_ and 55 vol% CH_4_ (stage A). At the 30th hour, the feed gas was replaced with the mixed gas containing 79 vol% CO_2_ and 21 vol% CH_4_ (stage B). Then at the 50th hour, it was restored to the mixed gas same with stage A (stage C). In the three stages of A, B, and C, the PPPS/PDMS/PSf composite membrane maintained stable CO_2_/CH_4_ separation performance, indicating that the composite membrane exhibited excellent short-to-mid-term stability.

### 3.5. Performance Comparison of PPPS/PDMS/PSf Membrane with the Commercial and Reported Membranes

Table 1 exhibited some commercial and reported membranes for CO_2_/CH_4_ separation. Compared with other membranes, the PPPS/PDMS/PSf membrane displays acceptable CO_2_ permeance and excellent CO_2_/CH_4_ selectivity, which has great potential for large-scale application in CO_2_ reuse and CH_4_ recovery in oilfield associated gas.

## 4. Conclusions

Compared with traditional separation methods such as cryogenic rectification and adsorption, the membrane separation process features operation flexibility, ease of scale-up, no phase change, and no need for regeneration. The membrane separation system is feasible to be designed as an integrated skid-mounted structure, which is convenient for transportation and movement in the remote wilderness. Therefore, membrane separation possesses obvious advantages in CO_2_ reuse and CH_4_ recovery in oilfield associated gas.

In this paper, the PPPS/PDMS/PSf composite membrane with proper thickness and defect-free composite layers was successfully prepared by 1.5 wt% PDMS and 0.15 wt% PPPS, which exhibited high CO_2_ permeance of 3.451 × 10^−7^ mol·m^−2^·s^−1^·Pa^−1^ and CO_2_/CH_4_ selectivity of 62 at 298 K, saturated humidity and 0.15 MPa. The two-stage membrane separation process using PPPS/PDMS/PSf composite membranes was simulated by adjusting the feed gas CO_2_ concentration and pressure. When the 1st- and 2nd-stage pressures are 1.1 MPa and 0.3 MPa respectively, the minimum specific cost of the two-stage membrane separation process using the PPPS/PDMS/PSf composite membranes to separate CO_2_/CH_4_ (45/55 vol%) mixed gas can be controlled within 0.046 $·Nm^−3^ CH_4_. In addition, the PPPS/PDMS/PSf composite membrane shown outstanding short-to-mid-term stability, indicating the potential of industrial application in CO_2_ reuse and CH_4_ recovery in oilfield associated gas.

## Figures and Tables

**Table 1:** Performance comparison with other commercial and reported membranes.

Membranes	CO_2_ Partial Pressure/MPa	CO_2_ Permeance/×10^−7^ mol·m^−2^·s^−1^·Pa^−1^	CO_2_/CH_4_ Selectivity	References
Polyactive^TM^	0.3	2.831	12.9	[44]
Pebax^®^1657	0.3	0.037	18	[45]
Pebax^®^1657/PEG (50/50)	0.175	0.214	15	[28]
DNMDAm-DGBAmE-TMC	0.044	4.02	51	[31]
TR-PBO	0.2	6.492	14	[46]
rGO-PBOI	0.1	5.976	32.4	[47]
PIM1-9GO-AEDPPF	0.05	20.1	17.4	[48]
PEBA/MOF-801	0.05	0.075	51	[49]
PPPS/PDMS/PSf	0.0675	3.451	62	This work

## Data Availability

The data presented in this study are available in the article and the supplementary material.

## Supplementary Materials

The following are available online at https://www.mdpi.com/2077-0375/11/2/118/s1.
