# Supplementary material for: Efficient Facilitated Transport Polymer Membrane for CO2/CH4 Separation from Oilfield Associated Gas
Source: Membranes (Basel). 2021 Feb 7;11(2):118. doi: 10.3390/membranes11020118 (PMC7914511; doi:10.3390/membranes11020118)
Supplement: Supplementary file 1 [file membranes-11-00118-s001.pdf]

Supplementary Materials

S1. PSf support membrane information

The PSf ultrafiltration membrane used in this work was provided by Jozzon Membrane Technology Co., Ltd. (China). The molecular weight cut-off was about 45 kDa. The support membrane consists of non-woven fabrics (about 95 μm) and a polysulfone skin layer (about 30 μm). The surface image and skin layer morphologies of the support were shown in Figure S1. The average surface pore diameter was about 15 nm and the porosity was about 3.5%.

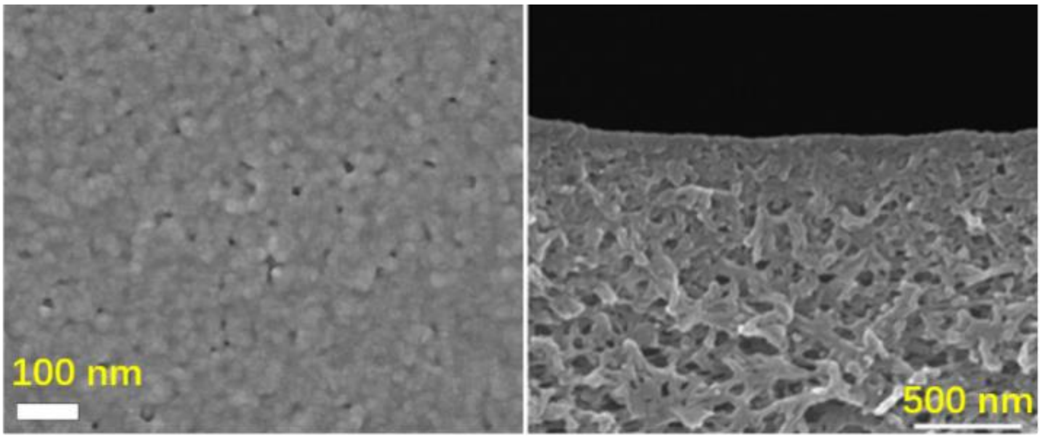

Figure S1. SEM surface and cross-section images of the support membrane.

S2. the mathematical models of cross flow

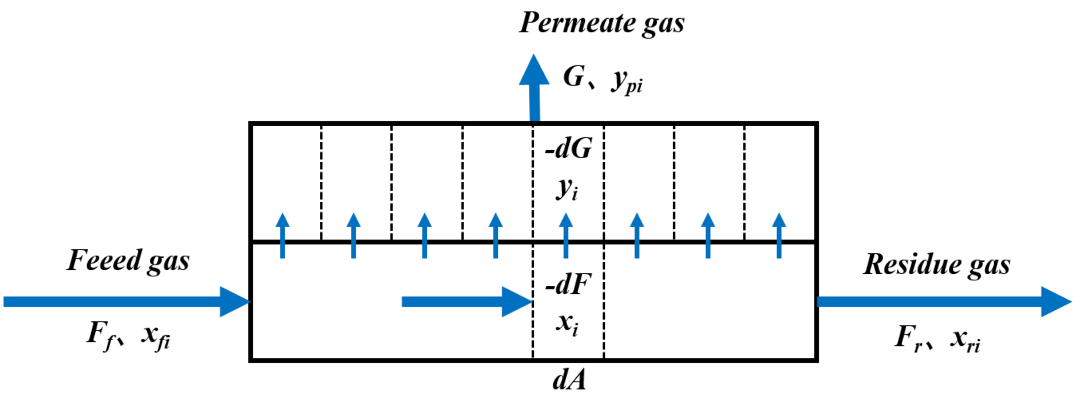

Figure S1. Schematic representation of cross flow in the spiral-wound membrane.

The gas separation process was described by the cross-flow model[1,2] and computed by MATLAB software according to previous work [3]. As shown in figure S1, the material balance equation at each differential area dA is shown below:

$$F_f = F_r + G$$

$$x_{fi}F_f = x_{ri}F_r + y_{pi}G$$

$$\frac{dF}{dA} = \sum_{i=1}^{n} R_i(P_H x_i - P_L y_i)$$

$$\frac{dx_i}{dA} = -\left[R_i(P_H x_i - P_L y_i) - x_i \sum_{j=1}^{n} R_j\left(P_H x_j - P_L y_j\right)\right]/F$$

$$\sum_{i=1}^{n} x_i = 1, \qquad \sum_{i=1}^{n} y_i = 1$$

In addition, the permeate side gas composition was obtained by

$$y_i = \frac{R_i(P_H x_i - P_L y_i)}{\sum_{j=1}^{n} R_j\left(P_H x_j - P_L y_j\right)}$$

Where F is the flow rate of the feed gas. R_i is the permeance of component i. x_i and y_i are the mole fractions of component i on the feed and permeate sides, respectively. P_H and P_L are the pressures on the feed and permeate sides.

S3. Estimation of investments and operating and maintenance costs

Table S1. The equations to determine the membrane process cost[3-6]

Items	Unit	Values
**Membrane**		
Total membrane area(S_m)	m^2	based on simulation data [5]
Membrane module cost (P_m)	$\$/m^2$	50
Reference frame cost (P_{mf})	$\$/2000\ m^2$	394000
Total membrane cost (I_m)		$S_m \times P_m$
Membrane frame cost (I_{mf})		$(S_m/2000)^{0.7} \times P_{mf}$
**Compressor**		
Feed gas flow rate of the compressors (Q_{cp})	Nm^3/s	based on simulation data
Energy consumption of the compressor (E_{cp})	kW	based on simulation data [5]
Compressor unit cost (K_{cp})	$\$/(Nm^3/s)$	96000 (0.3~0.9 MPa)<br>120000 (0.9~2.7 MPa)
Cost factor for housing, installation etc. (F_h)		1.8
Total compressor cost (I_{cp})		$Q_{cp} \times K_{cp} \times F_h$
**Expander**		
Energy consumption of the expander (E_{ex})	kW	based on simulation data [5]
Expander unit cost (K_{ex})	$/kW	500
Total expander cost (I_{ex})		$E_{ex} \times K_{ex} \times F_h$
**Heat exchanger**		
Reference heat exchanger (K_{he})	$\$/m^2$	300
Total heat transfer area (S_{he})	m^2	based on simulation data [5]
Total heat exchanger cost (I_{he})		$K_{he} \times S_{he}$
**Other parameters**		
The depreciation factor for commonly used equipment (d)		0.064*
The depreciation factor for the membrane (d_m)		0.225*
Operating time (t)	h/year	8000
Power cost (e)	$/kWh	0.1
Annual output of product gas ($V_{product}$)	$Nm^3/year$	based on simulation data
Capital cost (I_{cap})		$d \times (I_{cp}+I_{ex}+I_{he}+I_{mf})+ d_m \times I_m$
Annual operation and maintenance cost ($I_{O\&M}$)		$0.036 \times (I_{cp}+I_{ex}+I_{he})+0.01 \times (I_m+I_{mf})$
Annual energy cost (I_{en})		$t \times e \times (E_{cp}-E_{ex})$
Total annual cost (I_{total})		$I_{cap}+I_{O\&M}+I_{en}$
**Specific cost of product gas**	$\$/Nm^3$	$I_{total}/V_{product}$

*The lifetime of the membrane module is assumed as 5 years, and the lifetime of the membrane frame, compressors and expanders is assumed as 25 years.

References

1. Weller, S.; Steiner, W.A. Separation of gases by fractional permeation through membranes. *Journal of Applied Physics* **1950**, *21*, 279-283, doi: 10.1063/1.1699653.
2. Shindo, Y.; Hakuta, T.; Yoshitome, H.; Inoue, H. Calculation methods for multicomponent gas separation by permeation. *Separation Science and Technology* **1985**, *20*, 445-459, doi: 10.1080/01496398508060692.
3. Xu, J.; Wang, Z.; Qiao, Z.; Wu, H.; Dong, S.; Zhao, S.; Wang, J. Post-combustion CO_2 capture with membrane process: Practical membrane performance and appropriate pressure. *Journal of Membrane Science* **2019**, *581*, 195-213, doi: 10.1016/j.memsci.2019.03.052.
4. Qiao, Z.; Wang, Z.; Zhang, C.; Yuan, S.; Zhu, Y.; Wang, J.; Wang, S. PVAm-PIP/PS composite membrane with high performance for CO_2/N_2 separation. *AIChE Journal* **2013**, *59*, 215-228, doi: 10.1002/aic.13781.
5. Arias, A.M.; Mussati, M.C.; Mores, P.L.; Scenna, N.J.; Caballero, J.A.; Mussati, S.F. Optimization of multi-stage membrane systems for CO_2 capture from flue gas. *International Journal of Greenhouse Gas Control* **2016**, *53*, 371-390, doi: 10.1016/j.ijggc.2016.08.005.
6. Zhao, L.; Menzer, R.; Riensche, E.; Blum, L.; Stolten, D. Concepts and investment cost analyses of multi-stage membrane systems used in post-combustion processes. *Energy Procedia* **2009**, *1*, 269-278, doi: 10.1016/j.egypro.2009.01.038.
